# Role of microRNAs in Stroke and Poststroke Depression

**DOI:** 10.1155/2013/459692

**Published:** 2013-12-02

**Authors:** Han Yan, Min Fang, Xue-Yuan Liu

**Affiliations:** Department of Neurology, Shanghai Tenth People's Hospital, Tongji University School of Medicine, Middle Yanchang Road No. 301, Zhabei District, Shanghai 200072, China

## Abstract

microRNAs (miRNA), a sort of noncoding RNAs widely distributed in eukaryotic cells, could regulate gene expression by inhibiting transcription or translation. They were involved in important physiological and pathological processes including growth, development, and occurrence and progression of diseases. miRNAs are crucial for the development of the nervous system. Recent studies have demonstrated that some miRNAs play important roles in the occurrence and development of ischemic cerebrovascular diseases such as stroke and were also involved in the occurrence and development of poststroke depression (PSD). Herein, studies on the role of miRNAs in the cerebral ischemia and PSD were reviewed, and results may be helpful for the diagnosis and prognosis of cerebral ischemia and PSD with miRNAs in clinical practice.

## 1. Introduction

Stroke is a major refractory disease significantly threatening the human health and life with a high morbidity, disability, and mortality. In recent years, studies have reported that the incidence of stroke increased annually by 9% and has been a leading cause of death in China [1]. Poststoke depression (PSD) is one of the common complications of stroke and is characterized by mood abnormalities, self-blaming, sadness, and depression. PSD has been a major factor hindering the recovery of neurological functions and daily activities in stroke patients and is closely related to the social avoidance and increased mortality of stroke patients [2]. Recent studies reveal that microRNAs (miRNA) play an important role in the occurrence and development of stroke and exert regulatory effects on PSD. In this review, the regulatory roles of miRNAs in stroke and PSD are summarized.

## 2. Outline of miRNAs and PSD

### 2.1. Close Correlation of miRNA with Growth, Development, Differentiation, Physiological Function, and Diseases of the Nervous System

miRNAs are a group of single-stranded small noncoding RNA molecules with 19–22 nucleotides, which could posttranscriptionally degrade mRNA or inhibit the translation of mRNA via binding the untranslated region at 3′ end of target mRNA and furthermore influence the expression of target genes. miRNA genes are usually transcribed by RNA polymerases II and III. With the presence of enzyme Drosha, pri-miRNA is synthesized and then cut into pre-miRNA with hairpin by Drosha. In the cytoplasm, the pre-miRNA hairpin is cleaved into double-stranded miRNA by the RNase III enzyme Dicer. One of the mature single-stranded miRNAs is kept in the silencing complex and may cleave target mRNA via binding to the complementary 3′-untranslated region (3′-UTR) in the target gene, resulting in posttranscriptional silencing [3–5]. Since Rota et al. found the first miRNA (lin-4) in nematodes in 1993, more than 1400 miRNAs have been identified in mammalians [6]. miRNAs play important roles in the gene regulation and more than 50% of genes in mammalians are regulated by miRNAs [7]. Previous studies have demonstrated that miRNAs play important roles in multiple physiological and pathological processes. It has been shown that the functional disorder of miRNAs is closely related to some pathological processes of central nervous system including stroke, multiple sclerosis, temporal lobe epilepsy, Alzheimer's disease, schizophrenia, and bacterial meningitis [8].

Some miRNAs are associated with the development of the brain, differentiation of neurons, and advanced neurological functions (such as learning and memory) and play important roles in maintaining survival of mature neurons and regulating development and differentiation of neurons. miRNAs have been identified in zygotes, neural stem cells, and fetal brain [9]. The deficiency of miRNAs in the cerebral cortex and hippocampus may significantly influence the morphogenesis of neurons and brain [10]. Experimental introduction of miR-430 may partially reverse the abnormal morphogenesis due to miRNA deficiency [11]. miRNAs could regulate cell cycle and play crucial roles in the regulation of neuronal differentiation. MiR-132, -134, and -let-7 are also crucial for the formation and plasticity of synapses [12]. miR-1 and miR-133 may exert regulatory effects on the synaptic transmission at the neuromuscular junction [13]. In addition, miR-124a and miR-125b may also promote the outgrowth of axons [14].

Identification of the distribution and expression of miRNAs in the central nervous system is beneficial to investigating the role of miRNAs in stroke and PSD. To date, a lot of studies have been conducted to study the distribution and expression of miRNAs under physiological conditions and after cerebral ischemia. In physiological conditions, 152 miRNAs have been detected in different nervous tissues (central and peripheral nervous tissues) of rats [15]; the results showed 30 miRNAs were specifically expressed in the nervous tissues of rats and the expression of most miRNAs varies among sites of nervous tissues. For example, in the central nervous system, miR-21 is expressed in both cortex and olfactory bulb, but the expression of several miRNAs is confined to a certain nervous tissue (miR-let-7b, miR-16, miR-22, miR-206, and miR-143 are only expressed in the olfactory bulb). A series of miRNAs was closely related to the growth and development, differentiation, physiological function, and pathology of nervous system. These miRNAs together with their target genes were involved in some important processes of stroke including endothelial dysfunction, abnormal regulation of nervous system and blood vessels, formation of edema, cell apoptosis, inflammation and remodeling of extracellular matrix (ECM). Other studies also indicated that focal cerebral ischemia may significantly alter the transcription and translation of mRNA through the regulation of the expression of relevant miRNAs, which might be a pathophysiological mechanism underlying the stroke and PSD. These findings provide theoretical basis for the treatment of stroke and PSD.

### 2.2. Complicated Pathogenesis of PSD

The correlation between stroke and PSD is complex and PSD is one of common complications of stroke. At least 1/3 of patients with acute stroke develop depression [16]. Depression may increase the morbidity, disability, and mortality of stroke. In the chronic stage, the symptoms of depression may further deteriorate and finally result in recognition impairment. PSD may manifest as general emotional disturbance including anxiety, feeling of despair, and anhedonia. Studies have revealed that the pathogenesis of depression is attributed to the reduced neurogenesis and disturbance of brain plasticity. Timely use of antidepression therapy after stroke may significantly reduce the incidence of PSD. Rationale application of antidepressants may markedly improve symptoms of depression and provide neuroprotective effects to brain plasticity and neurogenesis. Accumulating pieces of evidence have shown that selective serotonin reuptake inhibitor (SSRI) could improve the prognosis of stroke patients and reduce the incidence of PSD. Although PSD is responsive to antidepressants, few animal models have been established for the investigation of depression, and the molecular mechanism underlying the pathogenesis of PSD remains unclear.

In recent years, some studies have been conducted to investigate the pathogenesis of PSD. In terms of the preventive effect of SSRI on PSD, serotonin theory is widely accepted. In this theory, the depression after acute stroke is attributed to the destruction of noradrenergic neurons and 5-HTergic neurons and related pathways resulting in the reduction of norepinephrine and 5-HT. The neurogenesis is closely related to the occurrence of PSD. It has been shown that cerebral ischemic rats presented depression, which could be alleviated by the increased neurogenesis in the hippocampus. Of importance, this alleviation could be induced by antidepressants, in which 5-HT may play an important role. Studies on stroke rehabilitation demonstrate that 5-HT deficiency could reduce the neurogenesis in the hippocampal dentate gyrus. As a neurotransmitter, 5-HT is involved in the neuronal plasticity via maintaining the synaptic communication between cortex and hippocampus. In addition, 5-HT is an initial signal in the differentiation of neuronal precursor cells into neurons. In vivo studies have revealed that 5-HT plays a crucial role in the neurogenesis of the hippocampus [17].

Mental stress may inhibit the synthesis of brain-derived neurotrophic factor (BDNF) in the hippocampus which may be a contribution to the occurrence of depression. All the anti-depressants may increase the synthesis of BDNF and its signal transduction in the hippocampus and frontal cortex. However, injection of BDNF into the dopamine pathway of mesolimbic system could elicit depression-like response. It is currently accepted that stroke may cause the reduction or even deficiency of BDNF, which is an important factor in the cause of PSD.

Other relevant factors including anatomic location of lesions, gene polymorphism, inflammatory cytokines, alteration of circadian rhythm, and social and psychological factors may also function in the occurrence of PSD. However, the specific relationship between these factors and PSD has not been confirmed [18–20].

## 3. Regulatory Role of miRNAs in Stroke

### 3.1. Upregulation or Downregulation of miRNAs in Stroke

In stroke, upregulation or downregulation of miRNA may be observed in the brain and serum. It was recently reported that miR-210 was closely related to hypozia and downregulation of miR-210 was detected in the plasma of stroke patients [21]. Moreover, the miR-210 level can also be used to predict the clinical outcomes of stroke patients. Tan et al. [22] detected the increasing expression of miRNAs in the serum of patients with ischemic stroke, and the pattern and level of miRNAs expression varied in different types of stroke. Some studies also found that miRNA expression was different between males and females, suggesting that miRNAs might be involved in the influence of gender on the response after stroke. Thus, we infer that miRNA expression is definitely changed after stroke, which may also be used in the diagnosis and prognosis of stroke patients. However, gender should be taken into account during the diagnosis and treatment of cerebrovascular diseases on the basis of miRNAs [23–25].

### 3.2. miRNAs Improve Stroke via Regulating Angiogenesis

Promoting angiogenesis in the ischemic region after stroke may increase the amount of capillaries and improve the focal circulation, which are important for the prognosis of stroke patients. Studies have shown that miRNAs play important roles in the regulation of angiogenesis [26]. van Solingen et al. [27] firstly confirmed that miR-126 could facilitate the angiogenesis following ischemia. Bonauer et al. [28] also found that miR-92a could significantly inhibit the angiogenesis in vivo and in vitro. In addition, there was evidence that showed that miR-21 may promote the proliferation of newly generated smooth muscle cells in the intima [29, 30] and downregulation of miR-222 could facilitate the migration and proliferation of endothelial cells, which may increase the angiogenesis in the plaques. miR-221/miR-222 family may regulate the new formation of blood vessels via the regulation stem cell factor receptor c-Kit [31, 32]. In terms of the important regulatory role of miRNAs in the angiogenesis, identifying miRNAs targeting the angiogenesis and further exploring the mechanism underlying the regulator role of these miRNAs in stroke are beneficial for the clinical treatment of stroke.

### 3.3. miRNAs Are Involved in Neuroprotection of Ischemia Preconditioning (IPC)

IPC has been widely accepted to attenuate brain injury after ischemia. However, the mechanism is still poorly understood. IPC refers to the adaptive tolerance to prolonged ischemia following recurrent transient ischemia [33]. Lee et al. [34] found in animal experiments that members in miR-200 and miR-182 family presented significant upregulation in the brain of rats undergoing IPC and subsequent focal cerebral ischemia (3 h). They also found that members in miR-200 family could downregulate proline hydroxylase (PHD2) to reduce neuronal death. In another study, miR-132 family was found to bind to methyl-CpG binding protein 2, which resulted in upregulation in itself and improvement of IPC [35]. These findings suggest that IPC may regulate the miRNA expression to activate neuroprotection related signaling pathways in case of ischemia, which then reduce the ischemic injury to neurons after stroke.

### 3.4. miRNAs Regulate Stroke and PSD via Stroke Mediated Inflammation

Immune mediated inflammation is involved in atherosclerosis. In stroke patients, the infiltration of inflammatory cells of the atherosclerotic plaques (such as monocytes, mast cells, and lymphocytes) in the arterial walls has been regarded as a marker of atherosclerosis. Stroke may initiate a series of inflammatory responses to promote the neuronal and endothelial death and induce the regeneration of astrocytes. In addition, miRNAs are closely related to the inflammation in stroke. Studies have shown that miR-155 was a target of inflammatory mediators, and miR-21 and miR-126 were also definitely associated with the inflammatory response after stroke. miR-125b upregulation may also inhibit the proliferation of astrocytes. miR-26a, -34a, -147, and let-7b may directly regulate IFN-*β* (an anti-inflammatory cytokine) in human and macaque to modulate the inflammatory response after stroke [36, 37].

After stroke, inflammatory cytokines are an important factor in the pathogenesis of PSD. Animal studies reveal that some miRNAs may activate relevant inflammatory cytokines which then activate serotonin transporter (SERT) and increase the 5-HT content in the brain. This may promote the occurrence and development of PSD to a certain extent.

### 3.5. miRNAs Regulate Stroke and PSD via Stroke Mediated Neurogenesis

The high mortality and disability following stroke are attributed to the apoptosis and necrosis of a large amount of neurons causing neurological dysfunction. Postischemic neurogenesis is a dynamic process and requires multiple growth factors and a series of signaling pathways. Giraldez et al. [38] investigate the regulatory role of miRNAs in the brain morphogenesis of zebrafish and found that knockout of Dicer could cause severe developmental defect of the brain morphogenesis, which suggests that Dicer plays important roles in the neurogenesis. In several studies, the biological functions of some miRNAs and their targets were identified. For example, miRNA-9 may act as a tumor suppressor gene to regulate the neurogenesis [39]. miR-124 is the most abundant miRNA in the brain. Studies indicate that the expression of miR-12a in the subependymal zone reduced significantly at 7 d after focal cerebral ischemia in rats [40]. In addition, after introduction of biological simulants of miR-124a into the neural progenitor cells in the subependymal zone, the proliferation of neural progenitor cells was significantly inhibited after ischemia, but their differentiation into neurons was facilitated. miR-124 may also negatively regulate signal transducer and activator of transcription 3 signaling pathway to promote the differentiation of embryonic stem cells into neurons, but the differentiation into astrocytes is inhibited. Thus, miR-124a might become a target in the promotion of endogenous neurogenesis after stroke.

Neurogenesis is closely associated with the recovery of behaviors in PSD. The newly generated cells in the hippocampal dentate gyrus increase significantly after stroke and may develop into new neurons, which is helpful to improve the learning and memory of patients with stroke. After stroke, the neurogenesis increases, which may facilitate the migration of neuronal stem cells into the injured sites and replace the necrotic cells after stroke, leading to the improvement of neurological functions after stroke. In terms of specific roles in miRNAs in the neurogenesis, we speculate that miRNAs play regulatory roles in the occurrence and development of PSD.

### 3.6. Other Aspects on the Relationship between miRNAs and Stroke

Other aspects on the relationship between miRNAs and stroke have also been investigated. Free radical induced injury has been found to be involved in the ischemic injury in case of stroke. Superoxide dismutase (SOD) is one of the important antioxidases which may scavenge free radicals. Studies have revealed that miR-145 might be involved in the posttrancriptional regulation of SOD-2 and plays important roles in the oxidative stress induced neuronal injury after ischemia [41]. Cell apoptosis is an important pattern of cell death after stroke and plays important roles in the pathological processes following cerebral stroke. Some miRNAs (such as miR-497, -15a, and -21) have been found to be involved in the cell apoptosis after stroke. Besides apoptosis, miRNAs are also associated with brain edema after stroke.

## 4. Regulatory Role of miRNAs in PSD

### 4.1. miRNAs Play Important Roles in PSD

The change in miRNAs level and mutation of miRNAs or their target mRNA may cause or even promote PSD. The interaction between miRNAs and mRNA is altered in case of depression. miR-30e is a tumor suppressor gene related to Alzheimer's disease, and its polymorphism is closely related to the attack and clinical manifestations of depression [42]. miR-183 is closely associated with the circadian rhythm and its polymorphism may cause depression.

Zhou et al. [43] found that some miRNAs were downregulated or upregulated in the brain of Wistar rats treated with anti-depressants. Electroconvulsive shock (ECS) is a modality for the treatment of severe depression. Studies showed the neuronal activity in case of ECS altered the expression of 119 miRNAs in the hippocampus [44]. miR-16 may exert antidepression effect via influence on SERT, a target of SSRI. Experiments have shown that the overproduction of miR-16 may cause reduction in SERT in the spinal nucleus and increase the transmission of 5-HT to exert anti-depression effect [44]. These studies were conducted in patients with depression at early stage, and the role of miRNA in depression is required to be further studied.

### 4.2. miRNAs Regulate PSD via Signal Transduction

As mentioned previously, depression after acute stroke is attributed to the reduction in norepinephrine and 5-HT due to destruction of noradrenergic neurons and 5-HTergic neurons. miRNA may play important roles in the occurrence and development of PSD through upregulation of 5-HTergic signal transduction. miR-16 could also activate SERT to regulate the synthesis and transmission of 5-HT and subsequently modulate PSD. In experiments, injection of fluoxetine into the spinal nucleus to block SERT synthesis may increase miR-16 content via Wnt signaling pathway, and miR-16 further increases the 5-HT activity to exert anti-depression effect. miR-16 may also increase the miR-16 content in noradrenergic cells of the coeruleus to influence the SERT functions [45]. miR-96 may upregulate HT1B expression via binding to 3′-UTR of HT1B mRNA, and may further increase the mergence of HT1B and cell membrane to exert effects. In addition, miR-195 may regulate the activities of 5-HT2A and 5-HT4 to increase the uptake and transmission of 5-HT [46]. Taken together, the functional alteration of miRNAs may influence relevant signaling pathways to cause PSD.

### 4.3. miRNA May Regulate PSD via Influencing Brain Derived-Neurotrophic Factor (BDNF)

The synthesis of BDNF is regulated by multiple miRNAs. In experiments, results showed that antidepressant paroxetine (SSRI) may increase the intracellular miR-30a-5p [47]. Besides the binding of miR-195 to 5-serotonin receptors, miR-195 may also regulate the synthesis and activities of BDNF receptor and glutamate receptor [48]. Specific miRNAs may form feedback with BDNF to maintain the BDNF in a normal level. Pharmacologically increasing the BDNF level without blocking the synthesis of specific miRNAs may exert a better therapeutic effect on PSD.

The expression of some miRNAs (such as miR-22, -200b, -211, and -300) increases in PSD and these miRNAs may bind to CREB (a transcriptional factor related to brain plasticity) to exert anti-depression effect. The binding of miR-124 to CREB may regulate 5-HT dependent synaptic plasticity [49, 50]. BDNF may induce the synthesis of miR-132 to regulate neurogenesis. Thus, the interaction between miRNAs and BDNF plays important roles in the pathogenesis of PSD.

### 4.4. Other Aspects on the Relationship between miRNAs and PSD

Psychological factor is also an important factor that influences PSD. miR-192/194, -219 and -182 are found to regulate the expression of genes related to circadian rhythm, which may partially reverse PSD due to sleep disorder and mood disorder [51]. miR-let-7, -9, -26, and -30 in the hippocampus and miR-124, -132/212, -134, and -183 in the tonsil increase significantly in the presence of acute or chronic stress. It is assumed that these miRNAs play crucial roles in the pathogenesis of stress induced PSD [52]. The increase in miR-18 and miR-124a may downregulate the glucocorticoid receptor expression to simultaneously reduce miR-132 and BDNF, which then exerts regulatory effects on stress induced PSD [53].

## 5. Prospects in Gene Therapy of Stroke and PSD with miRNAs 

miRNAs have been found to be involved in multiple pathological processes after stroke, which provides a direction for the investigation of mechanisms underlying the pathogenesis of PSD and brings promise for the effective prevention, diagnosis, and therapy of stroke and stroke related complications. miRNAs play important roles in gene regulation. However, the regulatory network of miRNAs and their target genes is complex. The therapy of stroke and PSD targeting miRNAs may face the temporal and special specificity and the entry of drugs into central nervous system. To date, numerous animal studies have been conducted to investigate the gene therapy of stroke, but only a few experiments are undertaken to investigate the correlation between miRNAs and ischemic stroke, and more problems exist in the therapy of stroke targeting miRNAs. In addition, few studies are conducted to establish the animal model of PSD. A large amount of miRNAs and their target mRNAs have formed a complicated network. Thus, to elucidate which neural structures or mechanisms are involved in the occurrence and development of stroke and PSD and which miRNAs are involved in the regulation of these mechanisms is dependent on the comprehensive understanding of the role of different miRNAs in the stroke and the role of altered expression of these miRNAs in stroke. With the development of biomedical techniques, increasing studies are undertaken to investigate the targets of miRNAs, which not only is beneficial to elucidate the mechanisms underlying the occurrence and development of stroke and PSD but also provides theoretical evidence for the diagnosis and treatment of stroke and PSD.
